# Senescence-associated proteins and nitrogen remobilization in grain filling under drought stress condition

**DOI:** 10.1186/s43141-022-00378-5

**Published:** 2022-07-11

**Authors:** Zohreh Hajibarat, Abbas Saidi

**Affiliations:** Department of Plant Sciences and Biotechnology, Faculty of Life Sciences and Biotechnology, Shahid Beheshti University, Tehran, Iran

**Keywords:** Senescence, Chlorophyll degradation, Protease, Drought, Nitrogen remobilization

## Abstract

**Background:**

Plants use escape strategies including premature senescence and leaf reduction to cope in response to drought stress, which in turn reduces plant leaves and photosynthesis. This strategy allows the new generation (seeds) to survive under drought but, plants experience more yield loss during stress condition. The amount of damage caused by drought stress is compensated by the expression of genes involved in regulating leaf aging. Leaf senescence alters the expression of thousands of genes and ultimately affecting grain protein content, grain yield, and nitrogen utilization efficiency. Also, under drought stress, nitrogen in the soil will not become as much available and causes the beginning and acceleration of the senescence process of leaves.

**The main body of the abstract:**

This review identified proteins signaling and functional proteins involved in senescence. Further, transcription factors and cell wall degradation enzymes (proteases) related to senescence during drought stress were surveyed. We discuss the regulatory pathways of genes as a result of the degradation of proteins during senescence process. Senescence is strongly influenced by plant hormones and environmental factors including the availability of nitrogen. During maturity or drought stress, reduced nitrogen uptake can cause nitrogen to be remobilized from leaves and stems to seeds, eventually leading to leaf senescence. Under these conditions, genes involved in chloroplast degradation and proteases show increased expression. The functional (proteases) and regulatory proteins such as protein kinases and phosphatases as well as transcription factors (AP2/ERF, NAC, WRKY, MYB, and bZIP) are involved in leaf senescence and drought stress.

**Short conclusion:**

In this review, senescence-associated proteins involved in leaf senescence and regulatory and functional proteins in response to drought stress during grain filling were surveyed. The present study predicts on the role of nitrogen transporters, transcription factors and regulatory genes involved in the late stages of plant growth with the aim of understanding their mechanisms of action during grain filling stage. For a better understanding, the relevant evidence for the balance between grain filling and protein breakdown during grain filling in cereals is presented.

## Background

Plants use strategies including premature senescence and leaf reduction to cope with stress, which in turn lead to a reduction in canopy. These strategies allow the plants to continue growth and eventually produce reasonable grain yield under stress. Such plants show greater reduction in yield during stress. At senescence stage, plants remobilize nutrients from the source tissues (leaves) to the reproductive organs and in this process the nutrients are remobilized. Senescence is controlled by complex networks of biochemical, physiological, and molecular mechanisms [1]. In addition, plants cope with drought stress by decreasing current photosynthesis and initiating leaf senescence, inducing a series of complex metabolic changes. Extensive physiological events subject to severe stress lead to chlorophyll degradation and production of reactive oxygen species (ROS), and oxidation of proteins and lipids. ROS produced in chloroplasts and mitochondria during drought may eventually cause leaf senescence and decrease yield [2]. As leaves begin to senesce, chloroplasts are degraded and stromal enzymes are destroyed, eventually reducing photosynthesis [3]. Carbon and nitrogen are important sources that are released during the senescence process or remobilized to other growing parts of plants. Sugars (glucose and fructose) accumulate during stress and in naturally senesced leaves. Although the accumulation of hexoses in older leaves initiates or accelerates the senescence processes, this alone may not cause senescence, but some other factors such as a complex metabolic network (nitrogen) and environmental factors may also be involved [4].

Under drought stress, plants face nitrogen deficiency, eventually leading to senesced leaves. Senescence due to nitrogen deficiency increases nitrogen recycling and remobilization, higher or optimal concentrations of nitrogen cause leaf growth and stay green [5, 6]. Therefore, improving the efficiency of Nitrogen use efficiency (NUE) is important for crops under water shortage conditions. Water deficiency accelerates the senescence process in cereals, leading to an increased loss of leaf nitrogen and leaf chlorophyll and increasing lipid peroxidation, which is important for remobilization of nitrogen to the phloem. If the nitrogen uptake is very low during grain filling, the plant’s nitrogen demand cannot be supplied. This condition decreases cytokinin biosynthesis and leads to degradation of leaf protein. Amino acids secreted by protein degradation are transported to the grain through the phloem. Over 95% of grain proteins are composed of amino acids, remobilized to seeds after degradation of proteins in rosette leaves [7].

Leaf senescence by extensively degrading chloroplast proteins using increased expression or activity of serine, cysteine, aspartic, and metalloproteases in leaves provide the nitrogen needed for seed filling. Senescence is a prerequisite for the mobilization of not only nitrogen but also other important nutrients. Chloroplasts contain a major reservoir of reduced leaf nitrogen. Hence, nitrogen remobilization essentially involves the degradation of chloroplast proteins into different types of amino acids and eventually mobilize to growing seeds [8]. Phyto-hormones, transcription factors, cellular oxidation state, and source strength cause the reallocation of nutrients, leading to senescence in natural environments or stress. Among the different transcription factors implicated in leaf senescence regulation, NAC and WRKY transcription factors and FTSH and Lon proteases have been surveyed extensively in barley. In this review, we will focus on the regulation of plant senescence in using signaling proteins such as protein kinase and phosphatase, senescence associated proteins, cell wall degradation enzymes and nitrogen remobilization in response to drought stress.

### Main text

#### Leaf senescence in response to drought stress

A genotype with a stay green (SG) phenotype relies more on current photosynthesis and retains more leaf chlorophyll, enabling them to synthesize carbohydrates and produce assimilates during flowering as well as during seed development. At the onset or delayed senescence, a positive correlation between water use efficiency and final yield in sorghum, wheat, rice, and maize has been observed, allowing the plants to cope under terminal drought stress [9].

The effect of SG phenotype and the share of its photosynthetically active tissue in spikes under terminal drought stress is of great importance in cereals. Recent research has demonstrated the importance of spike photosynthesis and the source of assimilate for effective grain yield under stress [10]. Under stress, senescence causes the remobilization of carbon and nitrogen from vegetative tissues (leaves and stems) to seeds and accelerates the seed filling process. These events alter carbon and nitrogen metabolism and disrupt remobilization mechanisms, leading to sink-source disruption through a complex network of hormones and regulation of genes network [11].

Most of reprograming of the thousands of genes related to senescence were up and down-regulated with the onset of senescence [3]. Many hydrolytic enzymes were targeted to degrade proteins, nucleic acids, and lipids [3]. Nitrogen produced by enzymes that break down proteins and lipids are remobilized through growing leaves to growing seeds and nourishing tissues. This process occurs due to natural conditions at the terminal of the plant growth period or biotic and abiotic stresses [12]. Enzymes involved in protein breakdown are one of the most important proteins related to leaf senescence with an essential role in nutrient recycling, especially nitrogen. Studies of genes expression showed that cysteine proteases are among the most abundant proteases involved during leaf senescence. Reduction of protein degradation in senesced leaf sections of wheat can be achieved by treatment of cysteine protease inhibitors, indicating the involvement of cysteine proteases in senescence [13, 14]. Proteases and signaling proteins play key roles in nitrogen remobilization form senesced leaves to developing seeds under both normal and stress conditions.

#### Degradation and remobilization of plastid proteins in leaf senescence

Proteases and ubiquitin proteins are involved in degradation of proteins. An excellent example of describing the genetic background of drought resistance is a situation where there is a significant genetic compatibility in a wide range of environments and a high level of drought tolerance [15]. The history of nitrogen mobilization for remobilization from older leaves to growing seeds goes back to more than 30 years. Drought stress Rubisco degradation is enhanced due to nitrogen demand from reservoir sources during leaf aging [16].

Biochemically active proteases (especially acidic pH cysteine endopeptidases) are found in older leaves and are found in vacuolar lithic tissues [16]. Pervious study has showed that plastid proteolysis stems contain many proteases, including members of the Clp, FtsH, and Lon families, as well as complementary active aminopeptidases [17]. In addition, a series of experiments have shown that isolated chloroplasts incubated in light or dark can initiate Rubisco degradation, resulting in the hydrolysis of its large subunit [18]. Other studies have also shown that the breakdown of this protein and possibly other proteins by oxidative stress may be important to initiate protein catabolism [19, 20]. Transcriptome analysis of old barley leaves showed that serine and cysteine protease genes are abundant, including genes encoding proteins normally found in lytic vacuoles [21]. Proteins and proteases involved in leaf protein degradation are shown in (Table 1). It is probable that chloroplast-based proteases are important for (initiating) the degradation of thylakoid protein. In addition, proteases may provide substrates for complete destruction by autophagy.

**Table 1:** Genes and proteins involved in leaf senescence

Characterization of genes and proteins	Gene/protein name	Description	References
**Protein degradation**	HvSAG12	Cysteine protease	[22]
	Subtilase	Subtilases	[22]
	UBC5	Ubiquitin.E2	[22]
	RGLG2	Ubiquitin.E3.RING	[22]
	UBP5	Ubiquitin.ubiquitin protease	[22]
	HvPAP20	Cysteine protease	[22]
	HvPAP14	Cysteine protease	[22]
	ATG8	Autophagy	[22]
	RD21	Cysteine protease	[22]
	RGLG2	Ubiquitin.E3.RING	[22]
**Regulation of RNA transcription**	MYB	MYB-related transcription factor family	[22]
	AtMYB44	Leaf senescence and abiotic stress responses	[3]
	HvNAC013	NAC domain transcription factor family	[22]
	HvWRKY12	WRKY domain transcription factor family	[22]
	IAA16	Aux/IAA family	[22]
	AFO	C2C2(Zn) YABBY family	[22]
	HvNAC001	NAC domain transcription factor family	[22]
	HvNAC005	NAC domain transcription factor family	[22]
	CDF4	Leaf senescence and floral organ abscission by regulating abscisic acid and reactive oxygen species pathways in *Arabidopsis*	[23]
	DEAR4	A member of DREB/CBF family, in leaf senescence and stress response	[24]
	ANAC055 and ANAC019	An important role in ethylene senescence response	[25]
	*RD26*	Leaf senescence and stress response	[26]
	*ARF2*	Dark-induced leaf senescence and accumulates for the onset of senescence	[27]
	*REVOLUTA (REV)*	Inset of leaf senescence in mature leaves	[28]
**Senescence-related proteins**	SAG14	Blue copper-binding protein (membrane)	[29]
	ACS6	ACC synthase	[29]
	SAG21	Late embryogenesis-abundant gene	[29]
	*SAG113*	Regulates stomatal movement and water loss in senesced leaves	[6]
	*SAG12*, 21, 29	Expression only occurs during the senescence of older leaves	[28]

#### Roles of cell wall degradation enzymes in leaf senescence

The cell wall is a complex and selectively permeable layer surrounding the cell. The most relevant function of the cell wall is to provide protection to the cell structure. Thus, the cell wall has evolved to be highly resistant to a wide range of biotic and abiotic stresses. The composition of plant cell walls is mainly composed of polysaccharides arranged in complex structures, while structural proteins are sub-components. The complexity of the cell wall is very important, because it is the first line of defense against biotic and abiotic stresses. In this regard, various plant enzymes involved in cell wall degradation are called cell wall degrading enzymes. Cell wall-degrading enzymes such as β-1,3-glucanase, glycoside hydrolases, β-glucosidase, expansion, and protease play key roles in sucrose metabolism in leaf senescence [29]. These enzymes increase expression in leaf senescence to provide the glucose needed for grain filling through cell wall breakdown. Cell wall degradation enzymes are known as senescence associated proteins.

Glycoside hydrolases (cell wall degradation enzyme) play roles in different biological processes like cell wall metabolism, plant defense, signaling, starch degradation, and remobilization of storage reserves (glucose). Endo 2,4 beta xylanase, Alpha galactosidase, Alpha N arabinofuranosidase A, and Beta D glucosidases were increased in the tolerant genotype. The expression of starch and sucrose metabolism pathway genes like Beta D glucosidase, catalyzing the hydrolysis of the starch, in wheat is altered during drought stress at grain filling stage [30]. Beta D glucosidases are enzymes that hydrolyze cellulose resulting in the formation of glucose. How these enzymes attack starch granule to release glucans is not yet well known, and the pathway of the starch degradation depends on plant species and physiological conditions [31]. It has been reported that cell wall hydrolytic enzymes have many different functions, implicated in stem remobilization during seed filling [31]. We have suggested that the cell wall degradation enzymes play a key role in carbon remobilization during seed filing in the tolerant genotype under drought stress.

#### Proteases involved in leaf senescence

Leaf senescence is a critical developmental step in plant’s life cycle. During this period, leaf cells affect intricate metabolic modifications and sequential degeneration in the cellular structure. These modifications contain several events namely, significant loss of chlorophyll (Chl), reduction of leaf photosynthetic potential, destruction of chloroplast structures, and the transition of nutrients to other sections of the plant, therefore leading to the death of the senescing leaves [32, 33]. The initiation of leaf senescence can be mainly controlled by different environmental components [34]. Several surveys have suggested that leaf senescence is a stage at which it initiates the programmed cell death (PCD) process [32, 34]. ATP-dependent protease complexes are great and consists of two large subunits of chaperone and proteolytic sections. These energy-dependent proteases along with chaperone system can play as quality control systems and are essential in the state of cell proteins in cell compartments such as cytosol, mitochondria, and chloroplasts [35]. These enzymes are expressed in germinating seeds and function in the remobilization of storage proteins to supply developing seedlings [12]. Most proteases involved in leaf senescence of many plant species are serine and cysteine proteases [12, 13]. Cysteine protease, SAG12, is a senescence-specific gene and is 900 times more pronounced by glucose. In addition, trehalose 6-phosphate, considered as a signal for the availability of high carbon, is needed for the onset of leaf senescence.

SAG2 and SAG12, both of which encode proteins associated with protein breakdown during senescence, are used as standard markers of leaf senescence. SAG2 transcription is regulated in leaves at all stages of growth, but increases during normal and stress-related senescence in leaves [36]. SAG12 is expressed exclusively in senesced leaves and the encoded protein is contained in the senescence-related vacuoles (SAVs). Senescence-related vacuoles are implicated in the degradation of chloroplast proteins like glutamine synthase and the large subunit of Rubisco [36]. SAG12 is one of the most widely used genes to detect leaf senescence. However, the role of this cysteine protease in nitrogen remobilization and leaf senescence process is not fully understood. The role of SAG12in nitrogen remobilization has not been well explored, and the few available reports are somewhat controversial. Although, *Arabidopsis* mutant sag12 did not show any phenotype, overexpression of OsSAG12-1 and OsSAG12-2 in rice moderate senescence progress [37]. In the withering processes, genes involved in senescence such as senescence-associated genes (*SAG*s), i.e., *SAG12* [38, 39], *SAG13* [40], were upregulated. Senescence associated proteins are involved in protein degradation, providing essential nitrogen during seed filling.

**Fig. 1:**
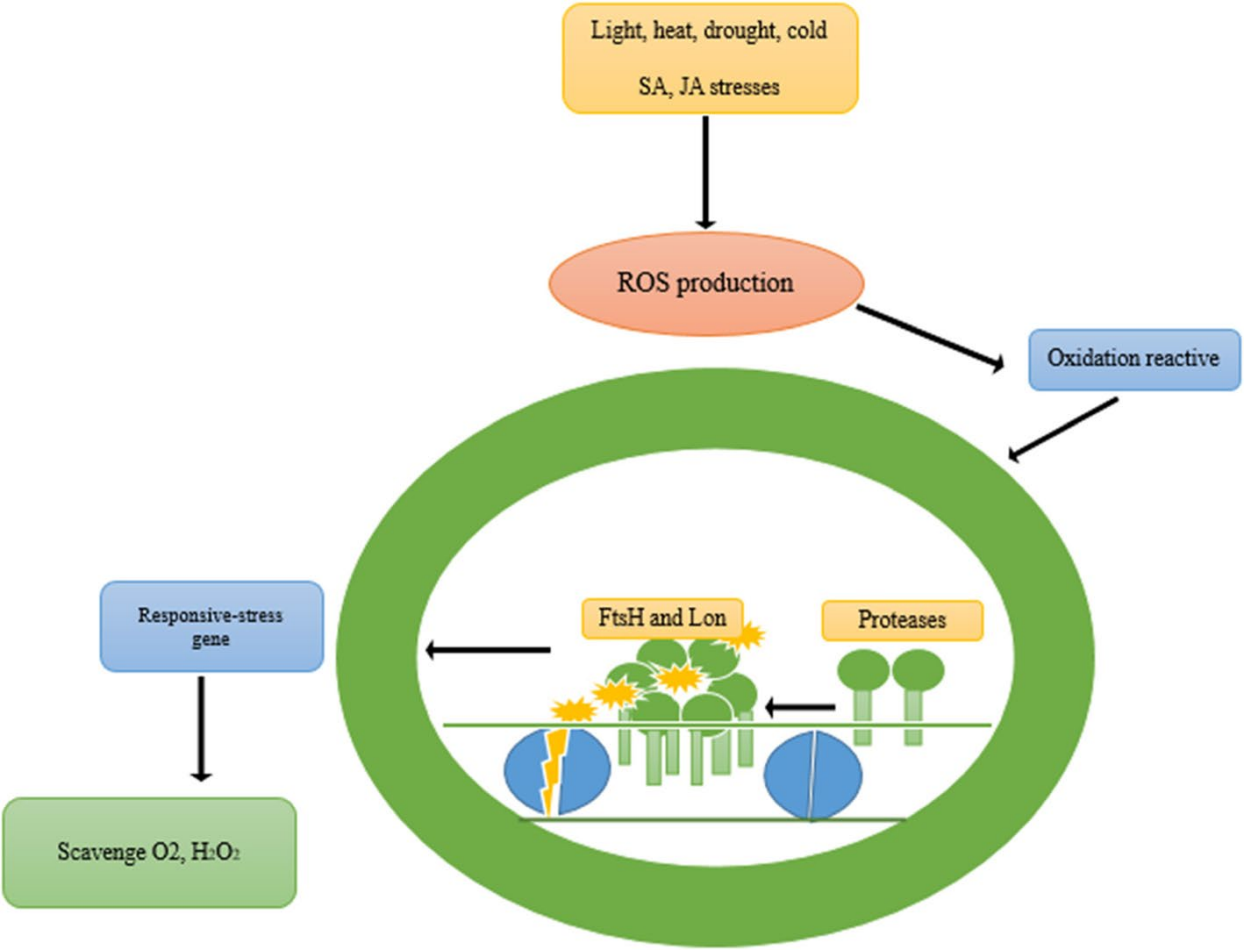
Stress-induced damage to PSII and removal of the damaged D1 by proteases. These stresses induce ROS production leading to oxidation reactive damages. Plants have evolved adaptive systems like responsive genes, protecting plants against oxidative stress damages. Also, FtsH and Lon repair D1 can assist in quality control of damaged proteins

When plants are exposed to stress conditions, the reaction protein D1 of the PSII is damaged by the ROS species produced near PSII, therefore leading to a reduction in PSII activity. Gene expression of different FtsH has been reported in senescent leaves of *Arabidopsis*. In *Arabidopsis*, FtsH2 was expressed most abundantly while FtsH1 and FtsH5 were downregulated in dark induced leaf senescence. In rice, FtsH2 and DegP play key roles in the regulation of D1 protein turnover and repair PSII cycle through ABA mediated leaf senescence [36]. Mitochondrial Lon and FtsH proteases are implicated in the biogenesis and maintenance of the oxidative phosphorylation system [41]. Mutation of ftsh6 was not able to degrade Lhcb3 and Lhcb1/3 during dark-induced senescence and high-light stress condition, respectively [42]. In oilseed rape, FtsH8 was upregulated under nitrogen deficiency at senescence stage. Whereas, in *Arabidopsis* and barley, Ftsh1, 5, and 6 were upregulated under light deficiency (dark) at senescence stage [42]. The main functions of protease include quality control systems in different organelles, degrade oxidative damaged proteins, and the biogenesis and maintenance of the oxidative phosphorylation [41]. These findings suggest that proteases can be used as important regulatory proteins for senescence in biotechnology systems. Although little information is available about their significance for leaf senescence, proteases have been shown to increase tolerance to abiotic stress conditions. Ftsh, Lon, and proteases are implicated in various stresses (light, heat, drought, cold and JA/SA) on protein degradation (Fig. 1).

#### Nitrogen remobilization after degradation of proteins in senesced leaves

The use of nitrogen by plants involves several stages including absorption, mobilization, recovery, and re-mobilization. Plants are exposed to adverse environmental factors during their growth stages and cannot escape many conditions of abiotic and biotic stress. To cope with these environmental stresses and survive in changing environments, plants remobilize nitrogen and carbon from the phloem of senesced leaves to growing tissues and storage organs such as seeds. Plants can save and use the limited nutrients and energy, to grow and produce seeds [43]. Nitrogen remobilization from protein (plastid) degradation and amino acid metabolism in leaf cell senescence to the protein endosperm stages of cereals involves several short and long distance transitions. Membrane transporters are essential for the transport of amino acids or oligopeptides from vacuoles and chloroplasts. Recently, researchers observed that sucrose-mediated transporters from mesophilic cells to apoplasts in *Arabidopsis* play an important role in carbon metabolism [44]. Nitrogen remobilization increases plant competitiveness, especially in nitrogen-restricted conditions for agriculture. Nitrogen remobilization efficiency is high because it can reduce the need for nitrogen fertilizers. In crops, nitrogen remobilization after anthesis and during seed maturation is highly related to grain yield and quality [45]. In small grains such as wheat and rice, up to 90% of the grain nitrogen content is remobilized from parts of the vegetative plant, while the proportion of nitrogen remobilization in maize is approximately 35–55% [46]. With the onset and progression of senescence or due to drought stress, several genes associated with senescence are upregulated. Under these conditions, in addition to a small current photosynthesis, plants break down proteins in the leaves and recycle and remobilize the protein to storage materials. Therefore, plants use complex molecular mechanism with functional proteins such as proteases and regulatory proteins such as phosphatase, protein kinase, and transcription factors to supply energy when the plant needs to produce seeds and offspring of the next generation. The NAC and WRKY transcription factor families play important roles in senescence-related gene regulatory networks in *Arabidopsis*, rice, and wheat [47–49]. The regulatory network of leaf aging in wheat and barley was investigated, where the transcription factors including NAC and MYB, WRKY, and AP2/EREBP played the most important role [49].

#### Senescence signaling and transcription factors in material remobilization under drought stress

During leaf senescence, the preferred forms of nitrogen remobilization are mostly in the form of amino acids and peptides with carbon remobilization. Researches on proteolysis and nitrogen remobilization during leaf senescence showed that the main remobilization stages include proteolysis of proteins such as rubisco to amino acids by proteases and their transport to the source organ. During the senescence process, proteins and genes associated with nitrogen transport and proteases were upregulated [50]. Nitrogen molecules can be transported from source by their transporters [51]. In wheat, a set of genes were upregulated in the early stages of leaf senescence (NF-YA) while the genes in the late growth stages included CAMTA, GRAS, and NAC. With senescence, bHLH transcription factors, TCP, and MADS_box, were downregulated mainly in the early stages of senescence. While GATA and MADS_box are involved in the later stages of senescence. Regulatory networks include various transcription factors that are themselves regulated by different phyto-hormones. A recent review of 48 barley NAC genes showed that many (but not all) of NAC genes increased expression in senesced barley flag leaf [51]. Among WRKYs, studies have shown that AtWRKY53 is a positive regulator of the senescence process [32, 40]. Limited information is available on the regulation of senescence by transcription factors for wheat and barley. The researchers found that when comparing transcription levels in a number of plant organs, HvWRKY23 expression was positively correlated with AtWRKY53 [52]. Thus, HvWRKY23 is involved in the regulation of leaf senescence. In particular, the mutant line was associated with a stay green phenotype and lower expression of WRKY53 and SAG12 during developmental stages [32].

Proteins involved in the regulation of aging through signal transduction (phosphorylation/dephosphorylation processes, ubiquitinite, and hormone signaling) are present in wheat and barley [53, 42]. Genes encoding protein kinases play a role in regulating flowering time. The functional characteristics of these genes are controlled by regulatory networks and are involved in regulation of senescence in cereals and may also use signaling pathways to control flowering time (Table 2). They provide free amino acids for nutrient recovery, where the nutrients are transported to the growing green tissues, either by feeding the metabolism of energy from the tricarboxylic acid cycle, or by providing cell metabolism as well as grain filling for seed production [54]. Study has shown that amino acids accumulate in dark due to senescence and nitrogen deficiency [55]. Several key gene families associated with drought stress were differentially expressed in this study, including TFs and reactive oxygen species scavengers [56]. Based on transcriptome analysis, most of 30 NAC genes increased expression during leaf senescence in *Arabidopsis*, revealing a strong relationship between hormones and environmental stress. ANAC072, ANAC019, and ANAC055 genes stimulate leaf senescence [57, 58]. A recent survey showed that *GhWRKY27* gene was upregulated in early stages of senescence and *its expression* varied in various cotton genotypes [59]. ANAC072 expresses the transcription of *CV* (chloroplast vesiculation), inducing a protein essential for protein degradation and the sugar transport gene *SWEET15* [60, 37]. Three WRKYs, namely WRKY18, WRKY25, and WRKY53, were upregulated in the onset and progression of senesced leaves [61].

**Table 2:** The transgenic plants developed by overexpressing the transcription factors

Gene name	Transgenic plant	Effect	References
GhWRKY91	*Arabidopsis*	Drought stress	[59]
OsWRKY23	*Arabidopsis*	Leaf senescence	[59]
GhWRKY42	*Arabidopsis*	Leaf senescence	[59]
GhWRKY27	*Arabidopsis*	Leaf senescence	[59]
TaWRKY10	Tobacco	Mediating osmotic balance, the production of reactive oxygen species (ROS)	[33]
*TaWRKY42-B*	*Arabidopsis*	Facilitates initiation of leaf senescence by promoting jasmonic acid biosynthesis	[62]
At*WRKY4*2	*Arabidopsis*	Leaf senescence through modulating SA and ROS synthesis	[63]
AtWRKY25	*Arabidopsis*	No accelerate senescence and tolerance to oxidative stress and the intracellular H_2_O_2_ level	[61]
*ZmVQ52*	*Arabidopsis*	Premature leaf senescence	[64]
*CpWRKY71*	*Arabidopsis*	Promotes flowering and leaf senescence	[65]
*GhWRKY17*	*Arabidopsis*	Accelerated leaf senescence	[59]
*NtNAC080*	*Arabidopsis*	Early senescence in leaves	[56]
TaNAC2	*Arabidopsis*	Enhanced tolerances to drought, salt, and freezing stresses, senescence	[66]
*OsNAC2*	Tobacco	Early senescence leaves	[30]
*AtNAC075*	*Arabidopsis*	Delays senescence in *Arabidopsis*	[67]
MlNAC5	*Arabidopsis*	Drought and cold tolerance and leaf senescence	[68]
*AtMYBL* (*At1g49010*),	*Arabidopsis*	as a putative novel transcriptional factor in leaf senescence, and responses to stress caused by exposure to ABA and salt	[69]
PheNAC3	*Arabidopsis*	Precocious senescence and abiotic stress tolerance	[70]
ScMYBAS1	Rice	Biomass accumulation and drought tolerance	[71]
MYBH	*Arabidopsis*	Leaf senescence	[72]
ANAC083 (VNI2), ANAC042	*Arabidopsis*	Tolerance to abiotic stresses, including heat and salt, and delays natural senescence negative regulator of senescence	[73]
GmNAC081	*Arabidopsis*	Enhanced senescence, a phenotype associated with accelerated leaf yellowing, reduced photosynthesis rate	[74]

A report at a transcriptomic level revealed that a higher number of DEGs were observed in the sensitive genotype (IS20351) than in the tolerant genotype (IS22330) [75]. This indicated that the susceptible genotype expresses more genes to complete its growth and life cycle whereas, the tolerant cultivar, instead of enhancing gene expression, stores its energy to complete its grain filling [75]. We can propose several key genes related to drought stress such as TFs, MAPK kinases, sucrose metabolism, starch degradation, and chaperones to be used in breeding and genetic engineering. The transgenic plants developed by overexpressing the transcription factors are given (Table 2).

#### Protein kinase and protein phosphatase in leaf senescence processes

Phosphorylation of protein kinases and phosphatases play an important role in cellular signaling. Some genes are up-regulated in signal transduction pathway such as protein kinases and calcium signaling pathway in leaf senescence. In bean (*Phaseolus vulgaris*), senescence-associated receptor kinase genes are up-regulated during leaf senescence [76]. Transcriptome analysis of leaf senescence in *Arabidopsis* showed that genes associated to the MAPK phosphorylation were upregulated [57]. For example, the membrane-bound receptor protein kinase RPK1 plays a key role in the regulation of leaf senescence in response to ABA. A MAP kinase kinase9 (MPK9) and MAP kinase kinase6 (MPK6) play significant roles in controlling leaf senescence [77]. In soybean, GmSARK gene and its *Arabidopsis* homolog, AtSARK, control leaf senescence in response to auxin and ethylene hormones [78]. It has previously been reported that a soybean kinase, GmSARK, and its *Arabidopsis* homolog, AtSARK, control leaf senescence through actions of auxin and ethylene [78]. Mutants of mkk9 or mpk6 can delay senescence, and overexpression of MKK9 gene causes premature senescence [77]. Other protein kinase, the membrane-bound receptor protein kinase RPK1 expressed by abscisic acid (ABA), can affect leaf senescence [79]. Sucrose-nonfermentation1-related protein kinase1 (SnRK1) is a negative regulator in the regulation of leaf senescence [80].

**Fig. 2:**
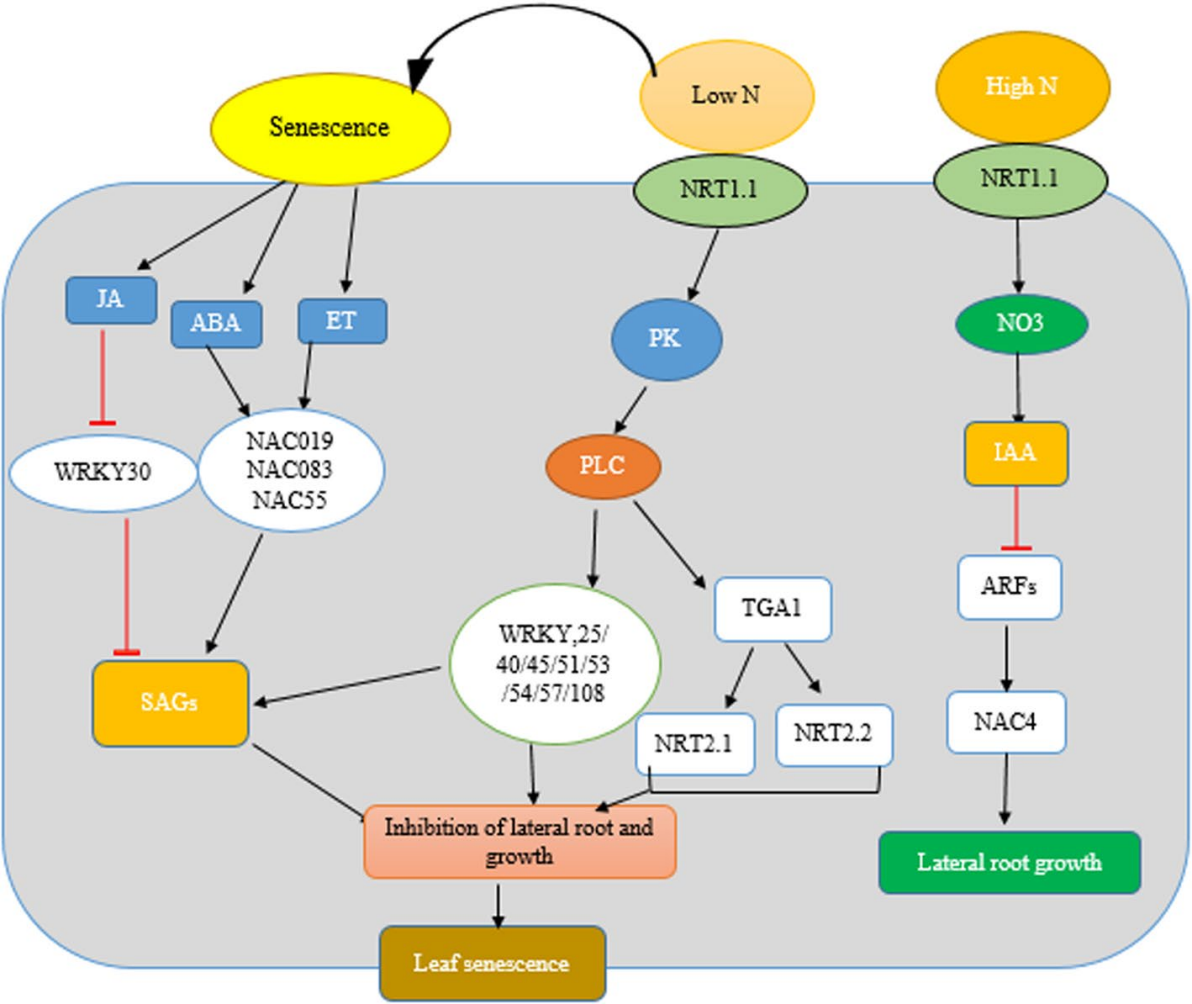
Schematic diagram showing the regulation of multiple pathways in response to high and low nitrate conditions in plants. Only the paths discussed in the present study are illustrated. Signaling pathways lead to the onset of leaf senescence. Black arrows indicate positive regulation of ABA; abscisic acid; IAA, indole-3-acetic acid; JA, jasmonic acid; SAGs, genes associated with senescence; TGA1; TGACG motif-binding factor, NRT, nitrate transporter; PLC, phospholipase C; ARF, auxin response factor transcription factors, and red lines indicate a negative signal as an inhibitory effect. Low nitrate and extremely low nitrate conditions have stimulatory and inhibitory effects on LR development, respectively, while high NO3 supply has an inhibitory effect on LR growth

Protein kinase and phosphatase can play positive and negative regulatory roles in senescence, but most of phosphatase proteins are regarded as negative regulators such as protein phosphatase 2C (PP2C) (Fig. 2). PP2C acts as senescence signaling, plant stress, and immunity [81]. Other protein phosphatases, senescence-suppressed protein phosphatase (SSPP), have negative regulatory roles in leaf senescence signaling [82]. A recent study showed that AtPR5K2 plays a key role in the relationship between ABA-dependent signaling and leaf senescence [83]. Some of the protein kinase and phosphatase proteins decreased or increased in response to leaf senescence. These proteins are important in senescence inhibition and are effective in yield increase.

#### Nitrogen homeostasis and senescence associated transcription factors

Under drought conditions, due to the lack of water, the availability of nitrogen in the soil is decreased and as a result, plants are exposed to stress, thus activating a number of molecular mechanisms in the plant. Due to drought stress, the onset of leaf and plant senescence is accelerated. Senescence makes it possible to induce transcription factors and nitrogen remobilization from protein breakdown. This process is a highly complex, regulated, and dynamic process, with some other possible side effects. Nutritional starvation, photosynthetic activity, carbon accumulation, carbon to nitrogen ratio, photoperiod, and various other symptoms can lead to the induction of aging of the organ and/or the whole plant. Previous studies have shown that leaf aging is a highly coordinated process, mediating redistribution of metabolites and reproductive maturity and ultimately leading to cell degradation [34, 84].

Environmental stresses such as drought and low nitrogen also cause premature leaf aging by altering gene expression and physiological activities. In addition, certain phyto-hormones, such as abscisic acid (ABA), ethylene (ET), jasmonic acid (JA), and salicylic acid (SA), have been shown to increase leaf senescence, while auxin may increase leaf aging (Fig. 2). ABA initiates and promotes leaf senescence, and as such ABA levels increase with increasing expression of ABA responsive genes during leaf aging [85, 86]. Under low nitrogen conditions, ABA, ET, and JA responsive genes cause inhibition of lateral root and plant growth, eventually leading to leaf senescence. Under high nitrogen conditions, auxin-responsive genes are activated which inhibit transcription factor ARF, leading to induction of root growth and root elongation, resulting in plant growth and development (Fig. 2). In older *Arabidopsis* leaves, more than 100 transcription factor (TF) genes from different families, including NAC and WRKY, have been upregulated, indicating that transcriptional regulation is an essential step in senescence process [85].

During the senescence process, genes associated with nitrogen transport and proteases show increased expression [50]. Inorganic nitrogen molecules can be transported from the leaves in the form of nitrate, ammonium, and urea by their respective transporters [51]. The NRT1.1, NRT1.7, and NRT2.5 transporters are involved in the remobilization of nitrate from source leaves to reservoir organs. The NRT1.6, NRT1.5, and AMT1.5 transporters are involved in nitrate and ammonium transport [86]. In wheat leaf aging process, a set of genes are increased in the early stages of leaf aging (NF-YA) while the genes in the late stages include CAMTA, GRAS, MADS_II, and NAC. With aging, bHLH-transcription factors, TCP, and MADS_box, are increased mainly during the aging stages. WRKY genes are a large TF group in the senescence transcript of *Arabidopsis* [87]. To date, functional analyzes have shown that several WFKY TFs from *Arabidopsis* positively or negatively regulate leaf aging [88]. For example, WRKY57 acts as a transcription factor for JA and auxin-mediated signaling in JA-induced leaf aging [87]. WRKY45 acts as a positive regulator of aging-induced leaf aging through a gibberellin-mediated signaling pathway (GA) [88]. WRKY25 negatively regulates leaf aging and acts as a positive regulator of WRKY53 expression whereas, WRKY30 negatively regulates leaf senescence [61].

As a dual affinity transporter, NRT1.1 can switch between high and low affinity in response to external nitrate concentrations through phosphorylation and dephosphorylation. When nitrate is low, NRT1.1 is phosphorylated, and converted to a high-affinity transporter, triggering a low-level nitrate response. When the nitrate concentration is high, NRT1.1 is dephosphorylated, and converted to a low-affinity transporter, causing a high-level nitrate reaction. Protein kinases interact with NRT1.1 and regulate nitrate signaling. As a positive regulator, protein kinase is specifically involved in low affinity responses, whereas negatively regulatory protein kinase is specifically involved by phosphorylating NRT1.1 in response to low nitrate high affinity responses [89]. Under low nitrate conditions, NRT1.1 can modulate auxin levels and meristem activity to suppress lateral root growth [90]. It also shows the phenotype of root formation in the presence of nitrate, possibly through dephosphorylation of the protein kinase to modulate NRT1.1, which indicates interference with the stress hormone ABA. A study has shown that NRT1.1 may be a sensor for root development [91], but its exact function in nitrate signaling is not yet fully understood. A close relationship between stress response and leaf senescence has been shown by the performance of several NAC- and WRKY family members [55]. For example, transcription factors such as NAC9 and NAC83 mediate osmotic stress signaling during leaf senescence [54].

To regulate gene expression, signals must be transmitted to the nucleus. Several transcription factors involved in the nitrate response have been identified. Among these, NAC transcription factors, TGA1, interact directly with nitrate-related target genes. Recent studies have identified the activation of transcription factors (TFs), including NAC (NAC019) and WRKY53 as key regulators of leaf aging and protein degradation [84]. Genes involved in hormonal signaling, i.e., abscisic acid (ABA) and ethylene, change during leaf senescence [92].

These factors are involved in the regulation of the degradation of chlorophyll, and NAC019 has been identified as a senescence activator in *Arabidopsis* [26 and 95]. Recently, a NAC factor in *Brassica napus*, NAC87, has been identified to regulate both ROS-regulating genes and leaf senescence [93].

#### Transcription factors involved in drought stress

Some of the main families of TFs involved in drought stress include AP2 / ERF, ARF, bZIP/HD-ZIP, C2H2, MYB/MYC, Zinc Finger, and NAC. WRKY TFs have key regulatory activity in growth processes and are also involved in the response to biotic and abiotic stresses [72]. Based on the number and similarity of AP2/ERF domains, the AP2/ERF family is classified into four main subfamilies: AP2 (Apetala 2), RAV (corresponding to ABI3/VP1), DREB (dehydration-responsive binding protein), and ERF. According to previous studies, the largest subfamily of TFs belong to AP2 / ERF. The ERF and DREB subfamilies have been shown to play an important role in responding to cold and dehydration stresses [24, 94].

DREB1 including CBF1, CBF2, and CBF3 play a major role in the response to cold/drought stress and in the aging process [77]. DREB1/CBF TFs specifically interact with DRE/CRT and express several non-living genes. They regulate stress responsiveness [95]. *DEAR*4 is a member of the DREB family and positively regulates leaf aging and response to multiple stresses in *Arabidopsis thaliana. DEAR*4 was identified as a regulator of cell death in the root region using the induced hyper-expression strategy. Plants, as sesile organisms, have developed complex mechanisms that are activated and integrated by expressing thousands of genes to cope with a variety of environmental stresses [96]. DREB2 proteins are involved in the response to dryness and heat [97].

Expression of OsDREB2A in rice was expressed under conditions of water scarcity and application of ABA, which can improve drought tolerance [97]. The OsDREB2B transcript was significantly increased under stress conditions and was able to increase drought tolerance [86]. As a result, OsDREB2 plays an important role in drought tolerance in plants. *Arabidopsis* finger zinc protein (AZF2), a C2H2 zinc finger TF, positively regulates leaf senescence. Recent studies have shown that ERFs are key regulators in abiotic stress tolerance in several species. Increased ERF expression after drought, salinity, cold, and heat treatments have been reported. For example, ERF1 is involved in the signaling pathways of ethylene and jasmonic acid. OsERF101 promotes leaf senescence through jasmonic acid signaling including OsNAP and OsMYC2 [54]. AtERF1 is considered a key component of the ethylene/JA-mediated defense response in *Arabidopsis* [98]. Activation of AtERF1 under various stress conditions, such as drought, salt, and heat, requires both ethylene and JA [98]. The SlERF5, a member of the AP2/ERF transcription factor family, acts as a transcriptional regulator and is a member of the ethylene responsive factor (ERF) family. The SlERF5 TF showed high expression in response to abiotic stresses, such as high salinity, drought, and cold temperatures [99]. OsERF71 positively regulates ABA signaling to alter root architecture and confer drought tolerance in rice [100].

The expression of *SlARF2A*, *SlARF2B*, *SlARF4*, *SlARF7A*, *SlARF8A*, and *SlARF9A* were significantly increased in tomato roots under drought stress [101]. *TaARF22* was upregulated in response to ABA treatment and showed a 60-fold change under drought stress [23]. ARF2 expression is induced in older leaves and regulates leaf aging and flower drooping [27]. A previous study reported that OsWRKY5 increases leaf aging by regulating ABA biosynthesis [95].

MYC2 was up-regulated in JA-induced leaf senescence by directly activating the expression of Senescence-Associated Gene 29 (SAG29). Abscisic Acid-Insensitive5 (ABI5), a leucine-based zipper (bZIP) of the TF type, was upregulated in ABA signaling and participated in enduring abiotic stress and leaf aging [102, 103]. In addition to MdABI5, MdBT2 was screened as a potential MdZAT10 interaction protein. MdBT2 plays a key role in regulating JA-mediated leaf senescence [104]. ATAF1, a positive senescence-regulated NAC TF, was upregulated by ABA, reactive oxygen species (ROS) treatment, and drought stress [92]. ATAF1 possibly induces ABA biosynthesis by interacting with the NCEDs (9-cis-epoxycarotenoid dioxygenases) promoter, which is a key regulatory in ABA biosynthesis [105].

The base leucine zipper family (bZIP) consists of a conserved bZIP domain [33]. In addition, bZIP TFs are involved in plant growth and in responding to abiotic stresses such as drought [106]. Members of the bZIP TF family have been isolated and identified in different eukaryotes. A study showed that OsbZIP23, one of the orthologous close to *Arabidopsis*, has been reported as a major regulator of ABA-dependent pathway [107]. The histone-binding protein-1b (HBP1b), belonging to the bZIP family of TFs, encoded by *SOBIC.009g23000* is induced in sorghum drought-resistant cultivars [106]. These TFs, expressed differentially in response to drought, are mostly responsible for drought stress differences among genotypes.

NAC016 is a positive regulator of aging and can enhance NAP and CCGs directly by binding to the promoter [108]. In *Arabidopsis*, drought tolerance increased with overexpression of three genes, namely ANACO19, ANACO55, and ANACO72. Similarly, overexpression of stress-responsive NAC1 (SNAC1) in rice provided tolerance to severe drought stress at the reproductive stage under field conditions without changing the phenotype [108]. In addition, OsNAC6/SNAC2 is caused by cold, drought, salinity, and ABA [107]. SlNAC6 i, a localized nuclear protein induced by abscisic acid (ABA) or polyethylene glycol (PEG), plays a positive role in tomato plant response to PEG stress [109–111]. Recent findings indicate the presence of various transcription factors inducing resistance in response to drought stress. In this article, we have surveyed various transcription factors that play important roles in drought stress and leaf senescence. Transcription factors play a central role in the aging process and plant defenses against pathogen attacks. In addition, TFs also participate in various processes such as drought tolerance and flowering.

## Conclusions

Herein, a favorable understanding of the mechanism and molecular signaling of remobilization from stem to grain under drought stress conditions has been discussed. We described the important roles of TFs, as a modern tool, to improve plant tolerance to multiple abiotic stresses. Identification of several stress-responsive TF genes and senescence-associated genes regulated by global stress responses or through signaling pathways of various hormones, including ethylene, JA, and SA, were evaluated. Taking all these observations into account, this study discusses the argument that AP2/ERF, NAC, and WRKY transcription factors certainly underlie hormonal signaling pathways that lead to leaf senescence in response to drought stress and play an important role in seed filling. Based on the available information, it can be said that during the terminal stages of growth and grain filling under plant stress conditions, tolerant genotypes can use the stored stem remobilization to supply the carbon and nitrogen needed by degradation of proteins in the leaves for grain filling. A balance should be designed and maintained between protein degradation and high yield towards the plant model along with protein degradation and stay green phenotype until the end of growth under drought stress. Furthermore, by increasing the expression of senescence-associated proteins such as transcription factors, protein kinase, phosphatases, and proteases, it is possible to improve the performance potential in response to stress. The relationship between senescence- associated proteins and nitrogen remobilization involves a complex network, where a balance between leaf senescence and nitrogen remobilization can provide a high quality and quantity of seed yield. One of the breeders’ objectives is to select cultivars with high nitrogen and carbon remobilization capacity, as well as genotypes with high photosynthetic potential. Researchers also need to understand the genetic basis of yield potential, physiological efficiency (biomass rate and aggregation of grain yield), and drought tolerance mechanisms to utilize effective strategies for genetic improvements of drought tolerant cultivars. Senescence is important at seed filling stage and is enhanced under drought stress. We reviewed transcription factors involved in drought stress and senescence. Significant progress has been made on the role of TFs in tolerating abiotic stress factors such as drought, and a significant number of TF genes have been identified and confirmed. Genetic manipulation of these stress-responsive TF genes is a powerful approach to improve plant tolerance. The present study discussed the significant contribution of transcription factors, transporters, and degrading proteins during senescence and their roles in drought stress response and grain filling. Senescence-associated proteins can be utilized in plant improvement programs.

## Data Availability

Not applicable.

## Abbreviations

ROS: Reactive oxygen species
SG: Stay green
PCD: Programmed cell death
SAGs: Senescence associated genes
